# Letter from W. A. Cusick, M.D., of Gervais, Oregon

**Published:** 1874-04

**Authors:** W. A. Cusick

**Affiliations:** Gervais, Oregon


Gervais, Oregon, March 3d, 1874.

*Editors Southern Medical Record:*

In looking over your January number, I see an article by Dr. Harris, of Greenesboro, Ga., "On the use of Veratrum Viride in Pneumonia and certain forms of Fever."

I think the Doctor's views as to the real nature of the difficulty, (the overwhelmed condition of the capillaries from excessive action of the heart) as well as his plan of management is excellent. I have used the veratrum viride in similar cases, and find that it will accomplish all that is claimed for it. There is but one thing in connection with its use, which I would have different. It is not sufficiently rapid in its action, where the abortive plan is attempted. In order to make an *immediate* impression, I have, for a year past, been in the habit of giving by hypodermic injection from ¼ to ⅓ of a grain of morphia sul., followed in from ten to fifteen minutes with ammonia carb., gr. iv.; ipecac, gr. ij., by the stomach. The ammonia prevents the morphia from affecting the head seriously, the morphia allays nervous excitement, thereby secondarily, quieting excessive action of the heart and securing rest to the overdisturbed capillaries, and a very profuse diaphoresis is the result—attended by a remarkable diminution in the frequency and force of cardiac action. After this is accomplished, I know of nothing superior to a combination of verat. viride, tr. hyoscyamin and spts. ether nit.

I have found the same plan to succeed equally well in our "autumnal" or "bilious remittent" fevers.

W. A. Cusick, M.D.

